# Changes of consultation-liaison psychiatry practice in Italian general hospitals: A comparative 20-year multicenter study

**DOI:** 10.3389/fpsyt.2022.959399

**Published:** 2022-10-14

**Authors:** Luigi Zerbinati, Laura Palagini, Matteo Balestrieri, Martino Belvederi Murri, Rosangela Caruso, Armando D’Agostino, Maria Ferrara, Silvia Ferrari, Antonino Minervino, Paolo Milia, Maria Giulia Nanni, Stefano Pini, Pierluigi Politi, Matteo Porcellana, Matteo Rocchetti, Ines Taddei, Tommaso Toffanin, Luigi Grassi, Italian Society of Consultation-Liaison Psychiatry, Jessica Bellucci, Emilio Bergamelli, Victor Attilio Campagna, Melissa Cherubini, Federica Folesani, Marta Gancitano, Francesca Giannetti, Gianluca Giovanna, Benedetta Gullotta, Lucia Massa, Giulia Montardi

**Affiliations:** ^1^Section of Psychiatry, Department of Diagnostic-Clinical Medicine and Public Health, University of Modena and Reggio Emilia, Modena, Italy, ^2^Dipartimento ad Attività Integrata di Salute Mentale e Dipendenze Patologiche, USL-IRCCS di Reggio Emilia, Reggio Emilia, Italy, ^3^Department of Health Sciences, Università Degli Studi di Milano, Milano, Italy; Department of Mental Health, ASST Santi Paolo e Carlo, Milan, Italy, ^4^Department of Neuroscience and Rehabilitation, Institute of Psychiatry, University of Ferrara, Ferrara, Italy, ^5^University Hospital Psychiatry Unit, Integrated Department of Mental Health and Addictive Behavior, University S. Anna Hospital and Health Trust, Ferrara, Italy, ^6^Psychiatric Operative Unit n.25 of Casalmaggiore (Cremona), “Oglio Po” General Hospital, Cremona, Italy, ^7^Department of Brain and Behavioral Sciences, University of Pavia, Pavia, Italy, ^8^Department of Clinical and Experimental Medicine, Psychiatric Clinic, University of Pisa, Pisa, Italy.; ^1^Department of Neuroscience and Rehabilitation, Institute of Psychiatry, University of Ferrara, Ferrara, Italy; ^2^University Hospital Psychiatry Unit, Integrated Department of Mental Health and Addictive Behavior, University S. Anna Hospital and Health Trust, Ferrara, Italy; ^3^Psychiatric Clinic, Department of Medicine, University of Udine, Udine, Italy; ^4^Department of Health Sciences, Università Degli Studi di Milano, Milano, Italy; ^5^Department of Mental Health, ASST Santi Paolo e Carlo, Milan, Italy; ^6^Section of Psychiatry, Department of Diagnostic-Clinical Medicine and Public Health, University of Modena and Reggio Emilia, Modena, Italy; ^7^Dipartimento ad Attività Integrata di Salute Mentale e Dipendenze Patologiche, USL-IRCCS di Reggio Emilia, Reggio Emilia, Italy; ^8^Italian Society of Psychosomatic Medicine, Parma, Italy; ^9^U.O. di Psichiatria, Dipartimento delle Fragilità, Azienda Ospedaliero-Universitaria, Sassari, Italy; ^10^Department of Clinical and Experimental Medicine, Psychiatric Clinic, University of Pisa, Pisa, Italy; ^11^Department of Brain and Behavioral Sciences, University of Pavia, Pavia, Italy; ^12^Department of Mental Health and Addiction Services, Niguarda Hospital, Milan, Italy; ^13^Department of Psychiatric Sciences and Psychological Medicine, University La Sapienza, 3rd Psychiatric Clinic, Rome, Italy

**Keywords:** liaison psychiatry, hospital psychiatry, medical psychiatry, Italy, Consultation-Liaison (C-L) psychiatry

## Abstract

**Introduction:**

Conducted under the auspices of the Italian Society of Consultation Liaison Psychiatry (SIPC) the aim of this study was to describe the characteristics of Consultation Liaison Psychiatry (CLP) activity in Italy (SIPC-2—2018) over the past 20 years by comparing with data from the first Italian nation-wide study (SIPC-1—1998).

**Methods:**

We collected data on CLP visits of 3,943 patients from 10 Italian hospitals over a period of 1 year. Data were compared with those from the SIPC-1 1998 study (4,183 participants). Patients were assessed with the same *ad hoc* 60-item Patient Registration Form recording information from five different areas: Sociodemographic, hospitalization-related, consultation-related, interventions and outcome.

**Results:**

Compared with participants from the previous study, SIPC-2-2018 participants were significantly older (*d* = 0.54) and hospitalized for a longer duration (*d* = 0.20). The current study detected an increase in the proportion of referrals from surgical wards and for individuals affected by onco-hematologic diseases. Depressive disorders still represented the most frequent psychiatric diagnosis, followed by adjustment and stress disorders and delirium/dementia. Also, CLP psychiatrists prescribed more often antidepressants (Φ = 0.13), antipsychotics (Φ = 0.09), mood stabilizers (Φ = 0.24), and less often benzodiazepines (Φ = 0.07).

**Conclusion:**

CLP workload has increased considerably in the past 20 years in Italy, with changes in patient demographic and clinical characteristics. A trend toward increase in medication-based patient management was observed. These findings suggest that the psychiatric needs of patients admitted to the general hospital are more frequently addressed by referring physicians, although Italian CLP services still deserve better organization and autonomy.

## Introduction

Consultation-Liaison Psychiatry (CLP), as the subspecialty of psychiatry liaising with other branches of medicine and stemming from the realization that some form of psychiatric comorbidity is evident in more than one third of patients admitted to the general hospital (GH) (1, 2), have grown considerably in many countries in the past decades (3). Appropriate CLP activity has positive effects in terms of length of hospital stay, health-related costs, and treatment adherence (4, 5).

This reflected a general interest in the CLP subspecialty at a European level, as evident from the involvement of international groups such as the European Consultation-Liaison Psychiatry Working Group (ECLW) (6) that led to the creation of the European Association of Consultation-Liaison Psychiatry and Psychosomatics (EACLPP) (7), further transformed into the European Association of Psychosomatic Medicine (EAPM) (8).

In Italy, the quest for efficient CLP models started after mental health legislation reform in 1978 with the development of CLP services within the GHs and CL activity only made by psychiatrists from psychiatric wards and some isolated University hospital experiences. More articulated CL services began in the 1990s in the form of integrated care programs and also highly specialized clinics. Pioneer examples were the services of Modena, Pavia and Udine for transplantation psychiatry (9–12), the psycho-oncology service of Ferrara and the psycho-gastroenterology service of Bari (13–15). In order to implement CLP in the country, a special CLP working group (CLP-WG.IT) within the Italian Society of Consultation-Liaison Psychiatry^1^ was created with the aim to promote a nationwide research project on a 1-year period to better analyze the CLP situation in Italy (16). That was the first and the only multicenter investigation in Italy. Ever since, Italian CLP has significantly changed, partly because of the development of new services as clinical CLP sub-specialization (17), but also for the change in the health systems, cutting of resources and “rationalization” of the way hospital care and needs of medically ill patients are delivered and managed for, with the risk to jump back to the past rather than improving the level of CLP.

Following the initiative of other European countries (18, 19), the need for an up-to-date examination of CLP activity in Italy was felt to be a priority (17). Therefore, the aim of this study was to evaluate the activity of CLP in Italy, highlighting the major changes happened in a 20-year time-span since the cited first CLP Italian study.

## Methods

The study aimed to compare data on current CLP activity in Italian GHs with those collected from the cited previous study by the CLP-WG-IT (16). To reach this aim, we used data from the nationwide CLP study conducted in 1998, referred to as Study 1, and the current CLP study conducted in 2018, referred to as Study 2.

Since we analyzed the data by using a form that is routinely employed in the CLP service after Study 1, ethical approval was operationalized by having each participant signing the information consent in agreement with the ethical regulations of the Committee for the Protection of Persons as adopted by the Local Health Trust and Hospital agencies of the participating centers.

### Study 1

The study was designed as a nationwide cross-sectional investigation involving 12 provinces (6 in the north of Italy, 3 in central Italy and 3 in southern Italy (Supplementary Figure 1), with a total of 17 GHs (19,804 beds overall) and 17 corresponding CLP services. The historical sample comprised 4,182 medically ill patients admitted to GH wards, recruited during a period of 12 consecutive months (1997–1998) for whom a psychiatric consultation was requested by hospital medical-surgical wards. A standardized Patient Registration Form (PRF-SF), previously used in CLP studies was used (16, 20, 21) to gather the following information: (1) Patient’s sociodemographic data, previous psychiatric history, use of psychiatric services and medications; (2) data related to the index hospitalization such as its length, time to referral (Lagtime1), the time between referral and consultation (Lagtime 2), type of ward and somatic diagnosis; (3) data related to the consultation (e.g., reason for referral, psychiatric diagnosis); (4) data related to the CLP intervention, such as psychopharmacologic prescriptions and transfer to other medical or psychiatric wards; (5) data related to patient outcomes, such as post-discharge plans, including the referral to outpatient psychiatric care. Psychiatric and somatic diagnoses were recorded using the WHO ICD-10 system.

### Study 2

The new CLP study, also run on a nationwide level, included 9 provinces, of whom 6 located in the Northern Italy, 2 in Central Italy, and 1 in Southern Italy (Supplementary Figure 1). A total of 10 GHs (8,338 beds in all) with a corresponding number of CLP Services were involved. From April 2018 to November 2019, CLP data were collected by using the same PRF-SF used in Study 1. Psychiatric and somatic diagnoses were also collected using the WHO ICD-10 system.

### Statistical analysis

Descriptive statistics were conducted using bivariate analysis. Differences in estimates between the samples were explored using *t*-test and Chi-square. To evaluate the magnitude of effect sizes we computed Cohen’s d, Kramer’s V and Phi (Φ) coefficient as measures of statistical robustness, while percentage difference (PD) and mean difference (MD) were calculated as measures of absolute differences between the variables of interest. Statistical analyses were performed using SPSS (Statistical Package for the Social Science)—20 package. To aid visual comparisons, appropriate figures and graphs were created using *ggplot* R package.

## Results

### Sociodemographic characteristics

In Study 2 data pertaining to 3,943 patients were collected and were compared to the sample of 4,183 patients of Study 1. The distribution of the consultations at both times according to the region of provenance are shown in Supplementary Figure 1; the two populations significantly differed according to the region of origin (Õ^2^ = 2791.75, df = 10, *p* < 0.001, V = 0.58).

Socio-demographic characteristics are shown in Supplementary Table 1. Compared with Study 1, patients in the present study were more frequently men (PD = 7.9%, Φ = 0.07) and older (MD = 9.98, Cohen’s *d* = –0.54). Data regarding age according to sex and the distribution of age according to psychiatric and somatic diagnoses are displayed in Supplementary Figures 2–4. Overall, Study 2 patients were less likely to be married (PD = 10.1%, Φ = 0.12) and more likely to be retired (PD = 6.4%, Φ = 0.06), unemployed (PD = 2.2%, Φ = 0.03) and living alone (PD = 4.6%, Φ = 0.06). Data regarding education level were not comparable between the two groups.

### Clinical characteristics

**FIGURE 1:**
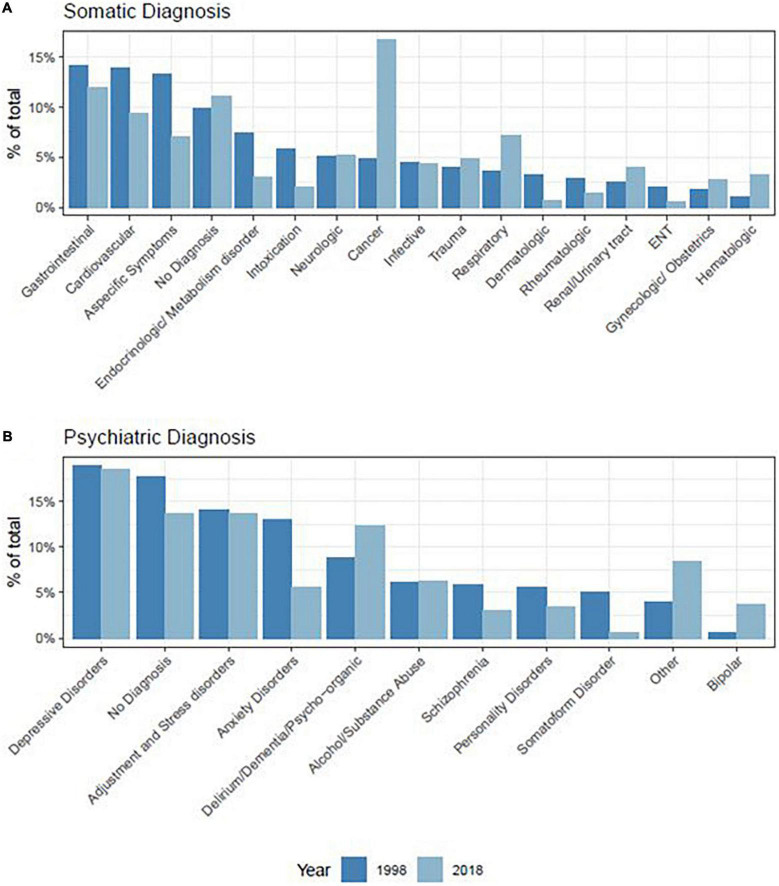
Percentage of somatic **(A)** and psychiatric **(B)** diagnoses of referred patients.

**FIGURE 2:**
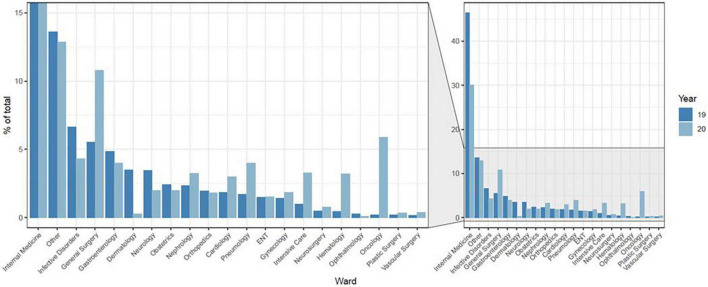
Distribution of referrals according to the ward.

Data pertaining to somatic diagnoses regarding referred patients are presented in Figure 1 and in Supplementary Table 2. Patients in Study 2 were more likely to suffer from cancer (PD = 12.8%, Φ = 0.20), hematological (PD = 2.3%, Φ = 0.07) or respiratory (PD = 3.9%, Φ = 0.08) diseases, and less likely to suffer from endocrine/metabolic disorders (PD = 4.4%, Φ = 0.09), dermatological conditions (PD = 2.5%, Φ = 0.08) or to show unspecified symptoms (PD = 5.9%, Φ = 0.09), including the effects of poisoning or intoxications (PD = 3.6%, Φ = 0.09). Figure 2 shows the distribution of referrals across different wards (see also Supplementary Table 3).

### Psychiatric consultation data

When comparing reasons for psychiatric referrals in Study 1 with Study 2, statistically significant changes were evident for the following reasons: Study 2 patients were more likely to be referred to psychiatric assessment for pharmacologic treatment (PD = 9.2%, Φ = 0.21), suicide risk (PD = 0.5%, Φ = 0.02), problems in patient’s management or compliance (PD = 2.1%, Φ = 0.05) or the presence of active psychopathological symptoms (PD = 23.1%, Φ = 0.20); they were also less likely to be referred for pregnancy issues (PD = 1.7%, Φ = 0.07), medically unexplained symptoms (MUS, PD = 6.6%, Φ = 0.13) and alcohol or substance abuse (PD = 1.6%, Φ = 0.04) (Table 1 and Supplementary Figure 5). Psychiatric referrals because of suicide attempt did not significantly differ between Study 1 and Study 2 (*p* > 0.05).

**TABLE 1:** Main reason for psychiatric referral.

Reason for referral (%)	1998	2018	Difference
Suicide attempt	4.9%	4.9%	Φ = 0.00, PD = 0%
Risk of suicide	1.0%	1.5%	*, Φ = 0.02, PD = 0.5%
MUS	9.3%	2.7%	**, Φ = 0.13, PD = 6.6%
Psychiatric evaluation	27.9%	0.6%	**, Φ = 0.38, PD = 27.3%
Patient management and compliance	2.3%	4.4%	**, Φ = 0.05, PD = –2.1%
Psychiatric symptoms	36.1%	59.2%	**, Φ = 0.23, PD = 23.1%
Psychopharmacologic consultation	0.5%	9.7%	**, Φ = 0.21, PD = 9.2%
Positive psychiatric history	3.8%	3.7%	Φ = 0.01, PD = 0.1%
Alcohol or substance problems	4.7%	3.1%	**, Φ = 0.04, PD = 1.6%
Abortion evaluation	2.4%	0.7%	**, Φ = 0.07, PD = 1.7%
Patient’s request	1.4%	1.0%	Φ = 0.01, PD = 0.4%
Other	5.2%	7.7%	**, Φ = 0.05, PD = 2.5%
Not specified	0.3%	0.6%	*, Φ = 0.02, PD = 0.3%
Patient informed about psychiatric referral	71.1%	83.5%	**, Φ = 0.14, PD = 12.4%

*p < 0.05; **p < 0.01.

PD, Percentage difference.

Patients were informed more often about psychiatric consultation referral in Study 2 with respect to Study 1 (PD = 12.4%, Φ = 0.14).

Both groups showed comparable rates of psychiatric care in the 5 years preceding the consultation (PD = 1.4%, *p* > 0.05, Φ = 0.00), although the pattern of mental health care in Study 2 patients was more prevalent in terms of psychiatric care at mental health outpatient services (PD = 5.4%, Φ = 0.07) and psychiatric private practice (PD = 7.5%, Φ = 0.12), and less prevalent at other services (PD = 2%, Φ = 0.03). Also, Study 2 patients reported a lower number of hospitalizations in psychiatric units in the previous 5 years (PD = 3.5%, Φ = 0.07) (Supplementary Table 4).

Time to CLP referral (Lagtime 1) was significantly higher in Study 2 (MD = 2.96, Cohen’s *d* = –0.23), while the time from referral to consultation (Lagtime 2) was significantly lower than in the Study 1 (MD = 0.57, Cohen’s *d* = 0.27). Study 2 patients received slightly fewer follow-up visits during the hospital stay (MD = 0.11, Cohen’s d = –0.11), with shorter consultation time (MD = 21.4, Cohen’s *d* = 0.43) and longer hospitalization length (MD = 4.67, Cohen’s *d* = –0.20) (Supplementary Table 5).

Supplementary Figure 6 shows the distribution of hospitalization length according to age, while the distribution of Hospitalization Length, Lagtime 1 and Lagtime 2 according to the psychiatric and somatic diagnosis are reported on the Supplementary Figures 7–12 and in Supplementary Tables 6–11.

Patients in Study 2 were more likely to be already treated with psychopharmacologic medications at the time of consultation than in Study 1 (PD = 17.7%, Φ = 0.17), especially with antidepressants (PD = 16.8%, Φ = 0.20), and mood stabilizers (PD = 8.9%, Φ = 0.19), but also with antipsychotics (PD = 8%, Φ = 0.11) and benzodiazepines (PD = 4.6%, Φ = 0.04) (Table 2 and Supplementary Figure 13A).

**TABLE 2:** Psychopharmacologic and liaison data.

	1998	2018	Difference
Psychopharmacologic treatment at the time of consultation	40.6%	58.3%	**, Φ = 0.17, PD = 17.7%
Patients on antidepressant treatment	12.4%	29.2%	**, Φ = 0.20, PD = 16.8%
Patients on benzodiazepines treatment	33.4%	38.0%	**, Φ = 0.04, PD = 4.6
Patients on antipsychotic treatment	11.2%	19.2%	**, Φ = 0.11, PD = 8%
Patients on mood stabilizer treatment	1.2%	10.1%	**, Φ = 0.19, PD = 8.9%
Psychopharmacologic prescription during the consultation	64.5%	75.9%	**, Φ = 0.12, PD = 11.4%
Antidepressant	27.2%	40.1%	**, Φ = 0.13, PD = 12.9%
Benzodiazepine	43.2%	36.0%	**, Φ = 0.07, PD = 7.2%
Antipsychotic	22.3%	30.5%	**, Φ = 0.09, PD = 8.2%
Mood stabilizer	0.4%	12.0%	**, Φ = 0.24, PD = 11.6%
Psychopharmacologic treatment at discharge	52.4%	46.6%	**, Φ = 0.05, PD = 5.8%
Antidepressant	27.9%	37.1%	**, Φ = 0.09, PD = 9.2%
Benzodiazepine	42.1%	29.8%	**, Φ = 0.12, PD = 12.3%
Antipsychotic	20.4%	24.9%	**, Φ = 0.05, PD = 4.5%
Mood stabilizer	0.5%	11.2%	**, Φ = 0.23, PD = 10.7%
Program at discharge/liaison intervention			
None	34.9%	38.6%	**, Φ = 0.03, PD = 3.7%
Social worker referral	3.2%	14.1%	**, Φ = 019, PD = 10.9%
GP referral	13.1%	0.0%	**, Φ = 0.25, PD = 13.1%
Psychiatric outpatient service	26.9%	20.3%	**, Φ = 0.07, PD = 6.6%
Consultation liaison service referral	13.2%	5.6%	**, Φ = 0.12, PD = 7.6%
Private practice psychiatrist	8.2%	14.5%	**, Φ = 0.07, PD = 6.3%
Other services (e.g., addiction clinics, child psychiatry service)	6.6%	11.9%	**, Φ = 0.09, PD = 5.3%
Psychiatric inpatient ward	2.9%	13.3%	Φ = 0.01, PD = 10.4%
Other medical ward	2.7%	1.7%	**, Φ = 0.03, PD = 1%

**p < 0.01.

PD, Percentage difference.

Regarding psychiatric diagnosis, it was more frequently made in Study 2 (PD = 2.5%, Φ = 0.03) and consisted of more frequent diagnosis of depressive disorder (PD = 1.8%, Φ = 0.02), bipolar disorder (PD = 3.5%, Φ = 0.11), behavioral syndrome due to delirium or dementia (PD = 4.9%, Φ = 0.07), and other disorders (PD = 5.4%, Φ = 0.11). Study 2 patients received less often a diagnosis of personality disorder (PD = 1.7%, Φ = 0.04), anxiety disorder (PD = 6.7%, Φ = 0.11), schizophrenia (PD = 2.5%, Φ = 0.05) or somatoform disorders (PD = 4.2%, Φ = 0.12), while adjustment disorder and substance abuse disorder did not significantly differ (all *p* > 0.05) between the 2 groups (Figure 1B and Supplementary Table 12).

### Intervention

Patients in Study 2 were more likely to be prescribed with psychopharmacological therapy (PD = 11.4%, Φ = 0.12) specifically, antidepressants (PD = 12.9%, Φ = 0.13), antipsychotics (PD = 8.2%, Φ = 0.09) and mood stabilizers (PD = 11.6%, Φ = 0.24) than Study 1, while benzodiazepines were less frequently prescribed (PD = 7.2%, Φ = 0.07) (Table 2 and Supplementary Figure 13B).

### Outcome

At discharge, Study 2 patients were less likely to receive psychopharmacologic treatment than Study 1 (PD = 5.8%, Φ = 0.05); overall in Study 2 antidepressants (PD = 9.2%, Φ = 0.09), antipsychotics (PD = 4.5%, Φ = 0.05), and mood stabilizers (PD = 10.7%, Φ = 0.23) were prescribed more frequently while the prescription of benzodiazepines was lower (PD = 12.3%, Φ = 0.12) (Table 2 and Supplementary Figure 13C).

Compared to Study 1, a therapeutic program at discharge was offered slightly less frequently in Study 2 (PD = 3.7%, Φ = 0.03), with patients being referred more often to social workers (PD = 10.9%, Φ = 019) and other specialized services (e.g., addiction clinics, child and adolescent psychiatric services; PD = 5.3%, Φ = 0.09) and less frequently to their general practitioners (PD = 13.1%, Φ = 0.25), psychiatric outpatient services (PD = 6.6%, Φ = 0.07), consultation-liaison services (PD = 7.6%, Φ = 0.12) and private practitioners (PD = 6.3%, Φ = 0.07). Psychiatric hospitalization rates did not differ between the two groups (*p* > 0.05), while in Study 2 patients were less frequently recommended to be transferred to other medical wards (PD = 1%, Φ = 0.03) (Table 2).

## Discussion

We report the results of a nationwide multicenter study describing CLP activity in Italy on almost four thousand patients, and comparing them to results from a previous CLP study adopting the same methodology of data collection carried out 20 years ago.

A first general result of the study is the increase of the ages of nearly a decade of the patients referred to CLP between the two studies. This finding seems to be in line with the general aging of the Italian population over the last 20 years and the increased life expectancy in Italy (22), as indicated in previous studies involving our centers (23, 24). This highlights the phenomenon of the general aging of the hospitalized population (25, 26), and the need of specific psychogeriatric training (27), since it has been estimated that up to 60% of old aged patients will develop a mental disorder during their hospital admission (28). Accordingly, this change had implications in the age of presentation of specific diagnosis and sub-populations of the sample (e.g., older patients with alcohol/substance abuse or personality disorders in Study 2), even though other differences can be attributed to specific epidemiological changes (e.g., the age of presentation of patients referred for infectious disorder in Study 2, while Study 1 coincided with the peak of HIV pandemic during the 90’s, with younger patients referred to CLP).

A second finding regards psychiatric referrals. Compared Study 1, patients in Study 2 were referred more often from surgical wards. A possible explanation might include a better understanding and more education about the importance of psychiatric variables by surgeon colleagues, as highlighted by increasing literature in the surgical field (29–33). Alternatively, the older population in Study 2 might have resulted in an increased number of referrals for behavioral problems, since about 80% of elderly patients undergoing surgery is expected to develop delirium (34). The latter explanation is in line with the modestly increased number of referrals from intensive care units and the higher number of referrals due to delirium, dementia or neurobehavioral syndromes, which, according to a recent nationwide Italian study, have been found to be present in 56.2% of the GH patients older than 65 (35).

Study 2 patients were also more likely to be referred from hematology-oncology settings. The almost quadrupled number of referrals for patients affected by these diseases reflects the important work done in the last two decades in the field psycho-oncology, with data showing a prevalence of psychiatric disorders in cancer care of 25–32% (36, 37). This is a major change of CLP activity in cancer setting that was shown to be lacking in Italy (38), while it seems to be a partially solved problem in more recent years (39).

A striking result is the lower rate of referrals for patients reporting MUS as the main reason for psychiatric evaluation and the low prevalence of somatoform disorders diagnosis in Study 2. There are different possible explanations for this finding. The first might be related to improvement in diagnostic tests (resulting in less “medically unexplained” symptoms, such as functional syndromes like fibromyalgia, functional gastrointestinal disorders and functional neurologic disorders), better understanding of these disorders, and improved education and management skills by non-psychiatrists, including rheumatologists, gastroenterologists and neurologists who have learned to treat common comorbidities like depression and anxiety (40). We cannot confirm this hypothesis though, since we do not have specific data in Italy. Another possible interpretation could be the older age of the sample. There is in fact evidence that MUS and somatic symptom disorders prevalence declines after the age of 65 years (41) and those older patients cope better with MUS than younger individuals (42), resulting in possibly less frequent referrals. Furthermore, since MUS in old-aged patients have been associated with frailty, the prevalence of these symptoms can be even lower because of misdiagnosis (43, 44). There is also the possibility that, given the complexity of the area, still with conflicts in the name and characteristics of the disorders and treatment, referring physicians may have less interest in requesting CLP consultation. This might be seen by referring physicians as only confirming the diagnosis but without concrete prospects of treatment in the usual organization of mental health service.

Regarding psychiatric diagnosis: the rates of adjustment and stress disorders, depressive disorders, and alcohol and substance abuse were comparable across the two studies. Depressive disorders still represent the most frequent psychiatric diagnoses, reflecting their high prevalence in the GH (45) and a general improvement in their recognition by non-psychiatrists (46). Similarly, the rates of adjustment and stress-related disorders, are comparable to other studies (47–50). The relatively high prevalence of alcohol and substance abuse disorders as reported in both the GH (51–54), and in CLP settings (55–57), highlight the fact that, at least in Italy, consultations for addictive disorders are still probably carried out by specific programs other than CL services. Anxiety disorders, compared with Study 1, were found to be significantly less prevalent, although with a similar rate found in other studies carried out in other countries (50, 58). This is consistent with a significant increase of antidepressants prescriptions in Italy during the last decades (59), as shown by the higher rates of patients being already in antidepressant treatment at the time of consultation. Patients may have already been prescribed antidepressants as outpatients, or by non-psychiatrists in the GH (60). Another possible explanation for this result again takes into account the older age of the population. It has been suggested that even though highly prevalent in old patients with chronic diseases (61), anxiety disorders might remain undetected in this particular population (62, 63). Bipolar disorders diagnoses showed a significant increase in prevalence at Study 2. This finding both contradicts (64, 65) and confirms (66) previous literature. Some hypotheses include the above-mentioned increase in antidepressant medications, with possible manic switching, and an improvement in the detection and diagnosis of bipolar disorders (67). Since it has been suggested that bipolar disorder can often be misdiagnosed as schizophrenia (68), this hypothesis might also explain the significantly decreased prevalence of schizophrenia and other psychotic disorders diagnoses in Study 2.

Further findings regard the length of stay, Lagtime1 and Lagtime2. Interestingly, while Hospitalization Length and Lagtime1 significantly increased in Study 2, Lagtime2 decreased. This last result probably indicates an overall improvement in the effectiveness of Italian CLP services, with psychiatric consultants able to deliver quicker visits. Increased Lagtime1 and hospitalization length can be interpreted in the light of the aging of the sample, since both age and Lagtime1 have been found to be predictors of Hospitalization Length (69–71) and, on the other hand, old age have been associated with increased Lagtime1 (69, 72). Patients displaying MUS may also require a higher number of investigations thus delaying the request of a psychiatric consultation (73). It should also be said that a still predominant tendency to consider CLP as a last resource, after all the possible medical investigations, can be found, with the need for implementation of proactive or integrated psychiatric care based services (3, 74).

Considering drug prescriptions, at the time of consultation patients were more likely to be already receiving psychoactive drugs, particularly antidepressants, mood stabilizers and benzodiazepines, compared with the previous study wave. CLP consultations resulted in an increase of antidepressants, antipsychotics and mood stabilizers prescription compared with 1998, and a relative decrease of benzodiazepines prescription. This pattern was relatively maintained at discharge, with a further decrease of benzodiazepines prescription in line with recent guidelines (75). With regards to mood stabilizers, the increase in prescription could reflect a change in their use for analgesic purposes as gradually emerged in medical literature (76–78). Overall, these findings mirror the aforementioned change in antidepressants prescription in the last decades, the increased prevalence of bipolar disorders and confirm the frequent use of benzodiazepines in medical settings both for sleep control as well as a means for rapid tranquillization (79). These data underline the need to further train non-specialists in psychiatry, within and outside the hospital, about the risks of benzodiazepines use in medically ill patients, especially in the elderly (80, 81), as well as the need to further develop CLP services with general practitioners to monitor pharmacotherapy (82).

Finally, interesting differences were shown in the type of consultation and liaison interventions. While in Study 2 there was only a very small decrease in liaison intervention at discharge, compared to Study 1 there were significant changes in referral patterns, with comparable rates of psychiatric inpatient admissions. A first issue comes from the increased referral to social work services, which could be explained by a grown awareness of the importance of the social component of the problems presented by patients, especially if affected by somatic diseases (83–85) and in times of recession and socio-economical crisis (86).

Regarding the post-discharge plan, patients in Study 1 were more often referred to specialized services (e.g., addiction clinics, child/adolescence psychiatric services, dementia clinics, eating disorders clinics, psycho-oncology services). This seems to support the implementation over the last 20 years of more special services within community psychiatry and an improvement of the organization of mental health services in Italy.

The study is strengthened by adequate sampling and nationwide participation. There are, however, limitations which should be mentioned. First, although there is a similar characterization and representation of the CLP services included in both Study 1 and 2, there are also differences in regional participation, such as a higher presence of Northern Italian centers in Study 2. Future studies should include a larger sample of centers and extend the research to a larger representation of GHs, including CLP activity in small community hospitals, which are under-represented in CLP studies in several countries (17). For these reasons, the generalizability of our results is not possible.

In conclusion, this study provides information about the current status of CLP in Italy. The data presented here confirm a predominant consultation-based approach to the psychiatric care of the medically ill patient in Italian GHs. The changes over time discussed in this article may support a more proactive approach in the provision of CLP services, and more consistent to relevant changes in the epidemiology of medical-psychiatric comorbidity; also, we hope they could guide future research on the topic and pave the way for structural changes in the delivery of care for the patient affected by psychiatric and somatic co-morbidities.

## Data availability statement

The datasets presented in this article are not readily available because of its proprietary nature. Requests to access the datasets should be directed to the corresponding author.

## Ethics statement

The studies involving human participants were reviewed and approved by the Ethical Committee for the Protection of Persons as adopted by the University Hospital and Hospital Local Health Trust of the participating centers. The patients/participants provided their written informed consent to participate in this study.

## Italian Society of Consultation-Liaison Psychiatry

The following persons participated as local coordinators of the Italian C-L Psychiatry working Group:

Jessica Bellucci^1,2^, Emilio Bergamelli^3^, Victor Attilio Campagna^3^, Melissa Cherubini^1,2^, Federica Folesani^4,5^, Marta Gancitano^4,5^, Francesca Giannetti^6^, Gianluca Giovanna^7^, Benedetta Gullotta^4,5^, Lucia Massa^8^ and Giulia Montardi^1,2^

^1^Section of Psychiatry, Department of Diagnostic-Clinical Medicine and Public Health, University of Modena and Reggio Emilia, Modena, Italy, ^2^Dipartimento ad Attività Integrata di Salute Mentale e Dipendenze Patologiche, USL-IRCCS di Reggio Emilia, Reggio Emilia, Italy, ^3^Department of Health Sciences, Università Degli Studi di Milano, Milano, Italy; Department of Mental Health, ASST Santi Paolo e Carlo, Milan, Italy, ^4^Department of Neuroscience and Rehabilitation, Institute of Psychiatry, University of Ferrara, Ferrara, Italy, ^5^University Hospital Psychiatry Unit, Integrated Department of Mental Health and Addictive Behavior, University S. Anna Hospital and Health Trust, Ferrara, Italy, ^6^Psychiatric Operative Unit n.25 of Casalmaggiore (Cremona), “Oglio Po” General Hospital, Cremona, Italy, ^7^Department of Brain and Behavioral Sciences, University of Pavia, Pavia, Italy, ^8^Department of Clinical and Experimental Medicine, Psychiatric Clinic, University of Pisa, Pisa, Italy.

## Author contributions

LG, LZ, MBe, and SF conceived the research. LZ, RC, MN, MR, MBa, AD’A, AM, MP, IT, LP, SP, PM, SF, and TT contributed to data collection. LZ carried out the statistical analysis and wrote the first draft. All authors contributed to the manuscript and approved the final version.

## Footnotes

1
www.sipc.eu
