# Supplementary material for: Changes of consultation-liaison psychiatry practice in Italian general hospitals: A comparative 20-year multicenter study
Source: Front Psychiatry. 2022 Oct 14;13:959399. doi: 10.3389/fpsyt.2022.959399 (PMC9614237; doi:10.3389/fpsyt.2022.959399)
Supplement: Supplementary file 1 [file Data_Sheet_1.ZIP › Supplementary Tables revised.docx]

**Table S1. Socio-demographic characteristics of the sample**

|  | 1998 | 2018 | Difference |
| --- | --- | --- | --- |
|  | | | |
| Sex ** , Φ=0.07 | | | |
| Male | 39.9% | 47.8% | PD=7.9% |
| Female | 60.1% | 52.2% | PD=7.9% |
| Age | 50.82 (±18.77) | 60.80 (±17.82) | ** , d=-0.54, MD=9.98 |
|  |  |  |  |
| Family Status | | | |
| Unmarried | 27.2% | 17.1% | **, Φ=0.12, PD=10.1% |
| Conjugated | 50.8% | 38.8% | **, Φ=0.12, PD=12% |
| Separated | 6.0% | 6.9% | Φ=0.01, PD=0.9% |
| Widowed | 15.0% | 11.5% | ** , Φ=0.05, PD=3.5% |
| Unknown | 1% | 25.8% | ** , Φ=0.36, PD=24.8% |
|  |  |  |  |
| Working Status | | | |
| Employed | 28.6% | 19.9% | ** , Φ=0.1, PD=8.7% |
| Housewife | 0.7% | 3.9% | ** , Φ=0.1, PD=3.2% |
| Retired | 35.6% | 42.0% | ** , Φ=0.06, PD=6.4% |
| Student | 3.7% | 1.6% | ** , Φ=0.06, PD=2.1% |
| Unemployed | 8.4% | 10.6% | ** , Φ=0.03, PD=2.2% |
| Other/Disabled | 20.3% | 3.5% | ** , Φ=0.25, PD=16.8% |
| Unknown | 2.7% | 18.4% | ** , Φ=0.26, PD=15.7% |
|  |  |  |  |
| Living Status | | | |
| Alone | 14.9% | 19.5% | ** , Φ=0.06, PD=4.6% |
| Original Family | 17.3% | 9.9% | ** , Φ=0.1, PD=7.4% |
| Institute | 2.1% | 2.8% | Φ=0.02, PD=0.7% |
| Friends/Flatmates | 2.9% | 2.4% | Φ=0.01, PD=0.5% |
| Partner | 21.6% | 4.9% | *, Φ=0.2, PD=16.7% |
| Unknown | 1.5% | 17.7% | *, Φ=0.28, PD=16.2% |
|  |  |  |  |

*p<0.05; **p<0.01; PD= Percentage Difference; MD= Mean Difference

**Table S2. Distribution of somatic diagnosis**

|  | 1998 | 2018 | Difference |
| --- | --- | --- | --- |
| No Diagnosis | 9.9% | 11.6% | **, Φ=0.02, PD=1.7% |
| Infective | 4.4% | 4.6% | Φ=0.00, PD=0.2% |
| Oncologic | 4.8% | 17.6% | **, Φ=0.20, PD=12.8% |
| Endocrinologic/Metabolism | 7.5% | 3.1% | **, Φ=0.09, PD=4.4% |
| Hematologic | 1.1% | 3.4% | **, Φ=0.07, PD=2.3% |
| Neurologic | 5.1% | 5.4% | Φ=0.00, PD=0.3% |
| ENT | 2.0% | 0.6% | **, Φ=0.06, PD=1.4% |
| Cardiovascular | 14.0% | 9.8% | **, Φ=0.06, PD=4.2% |
| Pneumologic | 3.6% | 7.5% | **, Φ=0.08, PD=3.9% |
| Gastroenterologic | 14.1% | 12.5% | *, Φ=0.02, PD=1.6% |
| Renal /Urologic | 2.5% | 4.1% | **, Φ=0.04, PD=1.6% |
| Gynecologic / Obstetric | 1.8% | 2.9% | **, Φ=0.03, PD=1.1% |
| Dermatologic | 3.2% | 0.7% | **, Φ=0.08, PD=2.5% |
| Reumatologic | 2.8% | 1.5% | **, Φ=0.04, PD=1.3% |
| Aspecific Symptoms | 13.3% | 7.4% | **, Φ=0.09, PD=5.9% |
| Trauma | 4.0% | 5.1% | **, Φ=0.02, PD=1.1% |
| Intoxication /Poisoning | 5.8% | 2.2% | **, Φ=0.09, PD=3.6% |

*p<0.05; **p<0.01; PD= Percentage Difference

**Table S3. Distribution of referrals across different wards**

|  | 1998 | 2018 | Difference |
| --- | --- | --- | --- |
| Internal Medicine | 46.4% | 31.4% | **, Φ=0.15, PD=15% |
| Gastroenterology | 4.9% | 4.2% | Φ=0.01, PD=0.7% |
| Nephrology | 2.3% | 3.4% | **, Φ=0.03, PD=1.1% |
| Hematology | 0.5% | 3.4% | **, Φ=0.10, PD=2.9% |
| Cardiology | 1.9% | 3.1% | **, Φ=0.04, PD=1.2% |
| Oncology | 0.2% | 6.2% | **, Φ=0.17, PD=6% |
| Pneumology | 1.7% | 4.2% | **, Φ=0.07, PD=2.5% |
| Infective Disease | 6.6% | 4.5% | **, Φ=0.04, PD=2.1% |
| Dermatology | 3.5% | 0.3% | **, Φ=0.11, PD=3.2% |
| Neurology | 3.5% | 2.1% | **, Φ=0.04, PD=1.4% |
| Neurosurgery | 0.5% | 0.8% | *, Φ=0.02, PD=0.3% |
| General Surgery | 5.5% | 11.3% | **, Φ=0.10, PD=5.8% |
| Orthopaedics | 2.0% | 1.9% | Φ=0.00, PD=0.1% |
| Vascular Surgery | 0.2% | 0.4% | *, Φ=0.02, PD=0.2% |
| Plastic Surgery | 0.2% | 0.4% | Φ=0.01, PD=0.2% |
| ENT | 1.5% | 1.6% | Φ=0.00, PD=0.1% |
| Ophtalmology | 0.3% | 0.1% | Φ=0.01, PD=0.2% |
| Gynecology | 1.4% | 1.9% | Φ=0.02, PD=0.5% |
| Obstetrics | 2.4% | 2.1% | Φ=0.01, PD=0.3% |
| Intensive Care | 1.0% | 3.4% | ** Φ=0.08, PD=2.4% |
| Other Wards | 13.6% | 13.5% | Φ=0.00, PD=0.1% |

*p<0.05; **p<0.01; PD= Percentage Difference

**Table S4. History of psychiatric care in the previous 5 years**

|  | 1998 | 2018 | Difference |
| --- | --- | --- | --- |
| Any psychiatric care | 44.7% | 43.3% | Φ=0.01, PD=1.4% |
| delivered by GP | 11.1% | 9.9% | Φ=0.02, PD=1.2% |
| delivered by outpatient service | 14.1% | 19.5% | **, Φ=0.07, PD=5.4% |
| delivered by private practitioners | 7.5% | 15.0% | **, Φ=0.12, PD=7.5% |
| Other services delivered ^a^ | 11.7% | 9.7% | * , Φ=0.03, PD=2% |
| inpatient treatment | 7.4% | 3.9% | ** , Φ=0.07, PD=3.5% |

1. e.g. addiction service, CL service

*p<0.05; **p<0.01; PD= Percentage Difference

**Table S5. Consultation data**

|  | 1998 | 2018 | Difference |
| --- | --- | --- | --- |
| Number of Consultations per Patient | 1.52 ± 1.56 | 1.41 ± 1.06 | **, d=-0.11, MD=0.11 |
| Total Time Spent for the Consultation (minutes) | 65.0 ± 58.2 | 43.6 ± 30.0 | **, d=0.43, MD=21.4 |
| Days of Hospitalization | 15.37 ± 18.0 | 20.04 ± 30.05 | **, d=-0.20, MD=4.67 |
| Lagtime 1 | 5.17 ± 9.95 | 8.13 ± 14.94 | **, d=-0.23, MD=2.96 |
| Lagtime 2 | 1.54 ± 2.28 | 0.97 ± 1.76 | **, d=0.27, MD=0.57 |

*p<0.05; **p<0.01; MD= Mean Difference

**Table S6. Days of Hospitalization according to psychiatric diagnosis**

|  | 1998 | 2018 |
| --- | --- | --- |
| None | 16.33±17.84 | 20.28±26.18 |
| Adjustment and stress disorders | 15.16±17.31 | 26.8±40.04 |
| Depressive disorders | 14.75±20.38 | 18.87±27.32 |
| Alcohol/Substance Abuse | 15.61±25.85 | 16.35±30.23 |
| Personality Disorders | 13.1±12.39 | 10.67±17.33 |
| Anxiety Disorders | 13.38±12.46 | 17.66±37.82 |
| Delirium/Dementia/Psycho-organic | 21.6±21.1 | 21.44±27.43 |
| Bipolar | 13.67±11.39 | 14.63±12.72 |
| Schizophrenia | 16.23±19 | 20.73±48.21 |
| Somatoform Disorder | 12.17±9.21 | 8.25±6.44 |
| Other | 13.7±12.87 | 21.41±32.68 |

**Table S7. Days of Hospitalization according to the somatic diagnosis**

|  | 1998 | 2018 |
| --- | --- | --- |
| None | 9.49±12.34 | 12.84±32.32 |
| Infective | 23.55±26.6 | 26.87±25.16 |
| Cancer | 22.98±20.32 | 22.85±26.5 |
| Endocrinologic/Metabolism Disorder | 15.69±11.78 | 15.21±15.39 |
| Hematology | 20.51±19.11 | 23.04±17.79 |
| Neurologic | 18.87±21.08 | 25.13±40.32 |
| ENT | 12.94±12.46 | 11.33±7.32 |
| Cardiovascular | 16.46±12.86 | 20.51±25.99 |
| Respiratory | 19.36±18.39 | 21.5±24.34 |
| Gastrointestinal | 14.73±15.07 | 19.39±29.9 |
| Renal/Urinary tract | 16.71±19.19 | 19.05±19.34 |
| Gynecologic/Obstetrics | 11.72±10.55 | 4.19±4.5 |
| Dermatologic | 13.33±18.36 | 22.9±35.49 |
| Rheumatologic | 17.01±15.93 | 18.29±12.34 |
| Aspecific Symptoms | 12.58±13.14 | 18.23±26.56 |
| Trauma | 21.44±36.01 | 37.43±60.71 |
| Intoxications | 9.92±24.09 | 5.83±6.61 |

**Table S8. Lagtime1 according to the psychiatric diagnosis**

|  | 1998 | 2018 |
| --- | --- | --- |
| None | 6.48 ±10.12 | 8.12 ± 13.11 |
| Adjustment and stress disorders | 5.57 ± 11.32 | 11.81 ± 20.82 |
| Depressive disorders | 4.21 ± 7.95 | 7.44 ± 13.88 |
| Alcohol/Substance Abuse | 4.8 ± 15.84 | 6.12 ± 15.4 |
| Personality Disorders | 5.07 ± 9.41 | 5.36 ± 11.51 |
| Anxiety Disorders | 4.22 ± 6.31 | 4.68± 7.71 |
| Delirium/Dementia/Psycho-organic | 7.43 ± 13.12 | 8.67± 12.57 |
| Bipolar | 3.19 ±3.88 | 4.93±8.5 |
| Schizophrenia | 4.06±9.52 | 3.8±6.77 |
| Somatoform Disorder | 4.08±5.02 | 12.19±40.78 |
| Other | 4.7±7.45 | 7.7±13.16 |

**Table S9.** **Lagtime1 according to the somatic diagnosis**

|  | 1998 | 2018 |
| --- | --- | --- |
| None | 2.79±6.03 | 4.22±10.93 |
| Infective | 10.38±19.93 | 9.89±13.34 |
| Cancer | 8.81±12.35 | 9.94±12.64 |
| Endocrinologic/Metabolism Disorder | 4.29±5.78 | 5.11±8.67 |
| Hematology | 7.83±15.91 | 9.04±11.33 |
| Neurologic | 6.23±12.39 | 10.24±24.74 |
| ENT | 4.12±5.04 | 4.11±4.51 |
| Cardiovascular | 5.49±6.8 | 9.12±17.12 |
| Respiratory | 6.26±9.28 | 7.79±12.53 |
| Gastrointestinal | 5.27±9.2 | 8.76±16.34 |
| Renal/Urinary tract | 8.46±20.04 | 6.65±10.25 |
| Gynecologic/Obstetrics | 3.04±4.53 | 2.42±2.8 |
| Dermatologic | 4.81±7.33 | 4.11±6.73 |
| Rheumatologic | 5.65±8.45 | 8.64±9.47 |
| Aspecific Symptoms | 3.72±5.7 | 8.3±16.83 |
| Trauma | 5.21±8.17 | 10.5±17.49 |
| Intoxications | 3.12±13.82 | 1.56±2.36 |

**Table S10. Lagtime2 according to the psychiatric diagnosis**

|  | 1998 | 2018 |
| --- | --- | --- |
| None | \| 1.53 ± 2.51 \| \| --- \| | 1.13 ± 1.99 |
| Adjustment and stress disorders | \| 1.64± 2.1 \| \| --- \| | 0.94 ± 2.04 |
| Depressive disorders | \| 1.46± 2.06 \| \| --- \| | \| 1.05± 1.6 \| \| --- \| |
| Alcohol/Substance Abuse | \| 1.58± 1.95 \| \| --- \| | \| 0.87± 1.47 \| \| --- \| |
| Personality Disorders | \| 1.42±2.6 \| \| --- \| | \| 0.61± 1.01 \| \| --- \| |
| Anxiety Disorders | 1.83± 2.57 | \| 0.99±1.83 \| \| --- \| |
| Delirium/Dementia/Psycho-organic | \| 1.3±2.64 \| \| --- \| | \| 0.77± 1.42 \| \| --- \| |
| Bipolar | \| 1.59±2.62 \| \| --- \| | \| 1.08 ±1.66 \| \| --- \| |
| Schizophrenia | \| 1.36 ± 1.88 \| \| --- \| | \| 0.73± 1.23 \| \| --- \| |
| Somatoform Disorder | \| 1.47± 1.78 \| \| --- \| | \| 1.07±1.52 \| \| --- \| |
| Other | \| 1.59±1.92 \| \| --- \| | \| 0.82±1.78 \| \| --- \| |

**Table S11.** **Lagtime2 according to the somatic diagnosis**

|  | 1998 | 2018 |
| --- | --- | --- |
| None | 1.16±1.75 | 0.67±1.43 |
| Infective | 1.13±2.3 | 1.28 ±1.96 |
| Cancer | 1.72±3.31 | 1.01±1.86 |
| Endocrinologic/Metabolism Disorder | 2.46±2.72 | 1.25±2.49 |
| Hematology | 1.3±1.96 | 1.04±1.79 |
| Neurologic | 1.7±3.15 | 1.34±2.64 |
| ENT | 1.2±1.37 | 1.36±2.92 |
| Cardiovascular | 1.92±2.5 | 0.85±1.35 |
| Respiratory | 1.31±1.67 | 0.97±1.62 |
| Gastrointestinal | 1.51±1.96 | 0.9±1.57 |
| Renal/Urinary tract | 1.41±1.91 | 0.96±1.52 |
| Gynecologic/Obstetrics | 1.5±2.08 | 0.57±1.34 |
| Dermatologic | 1.1±1.57 | 0.84±1.25 |
| Rheumatologic | 2.2±2.71 | 1.29±1.77 |
| Aspecific Symptoms | 1.48±2.22 | 1.2±2.03 |
| Trauma | 0.85±1.06 | 0.72±1.04 |
| Intoxications | 1.02±1.72 | 0.4±0.77 |

**Table S12. Psychiatric diagnosis**

| **Psychiatric Diagnosis at Discharge** | | **1998** | | **2018** | | **Difference** |
| --- | --- | --- | --- | --- | --- | --- |
| None |  |  | 17.8% |  | 15.3% | **, Φ=0.03, PD=2.5% |
| Adjustment Disorder |  |  | 14.1% |  | 15.3% | Φ= 0.01, PD=1.2% |
| Depressive Disorders |  |  | 18.9% |  | 20.7% | *, Φ=0.02, PD=1.8% |
| Substance Abuse |  |  | 6.2% |  | 7.0% | Φ=0.01, PD=0.8% |
| Personality Disorders |  |  | 5.6% |  | 3.9% | **, Φ=0.04, PD=1.7% |
| Anxiety Disorders |  |  | 13.0% |  | 6.3% | **, Φ=0.11, PD=6.7% |
| Delirium/Dementia |  |  | 8.9% |  | 13.8% | **, Φ=0.07, PD=4.9% |
| Bipolar Disorder |  |  | 0.6% |  | 4.1% | **, Φ=0.11, PD=3.5% |
| Schizophrenia |  |  | 5.9% |  | 3.4% | **, Φ=0.05, PD=2.5% |
| Somatoform Disorders |  |  | 5.0% |  | 0.8% | **, Φ=0.12, PD=4.2% |
| Other |  |  | 4.0% |  | 9.4% | **,Φ=0.11, PD=5.4% |

*p<0.05; **p<0.01; PD= Percentage Difference
