# Supplementary figures and images for: Changes of consultation-liaison psychiatry practice in Italian general hospitals: A comparative 20-year multicenter study
Source: Front Psychiatry. 2022 Oct 14;13:959399. doi: 10.3389/fpsyt.2022.959399 (PMC9614237; doi:10.3389/fpsyt.2022.959399)

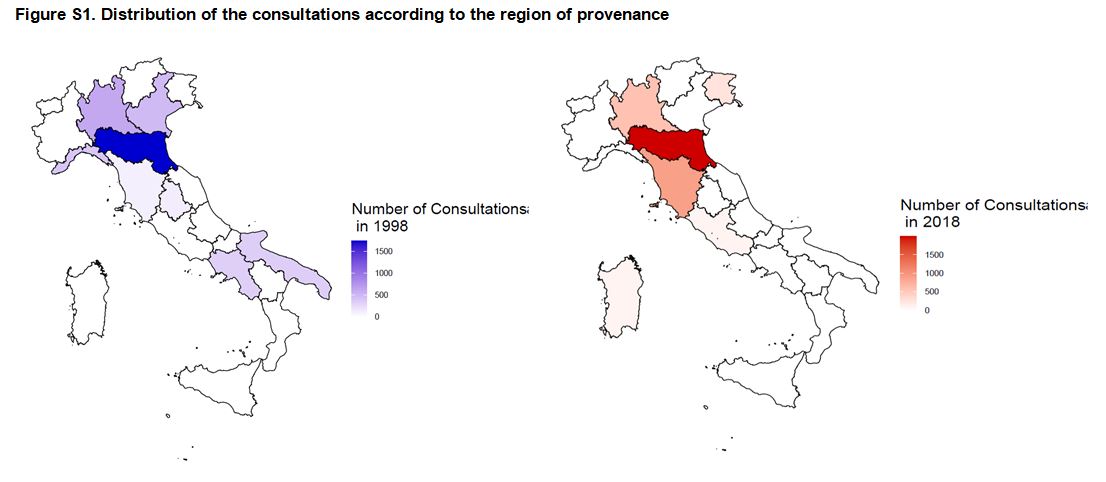

Supplement: Supplementary file 1 [file Data_Sheet_1.ZIP › Figure S1.JPG]

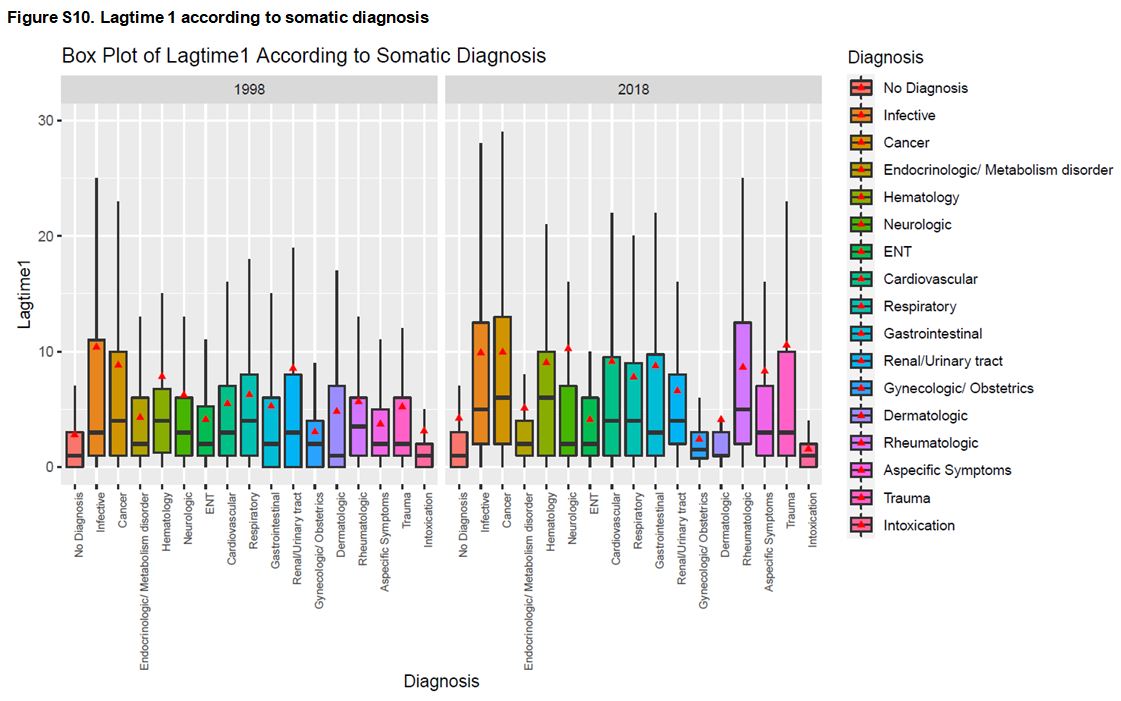

Supplement: Supplementary file 1 [file Data_Sheet_1.ZIP › FIgure S10.JPG]

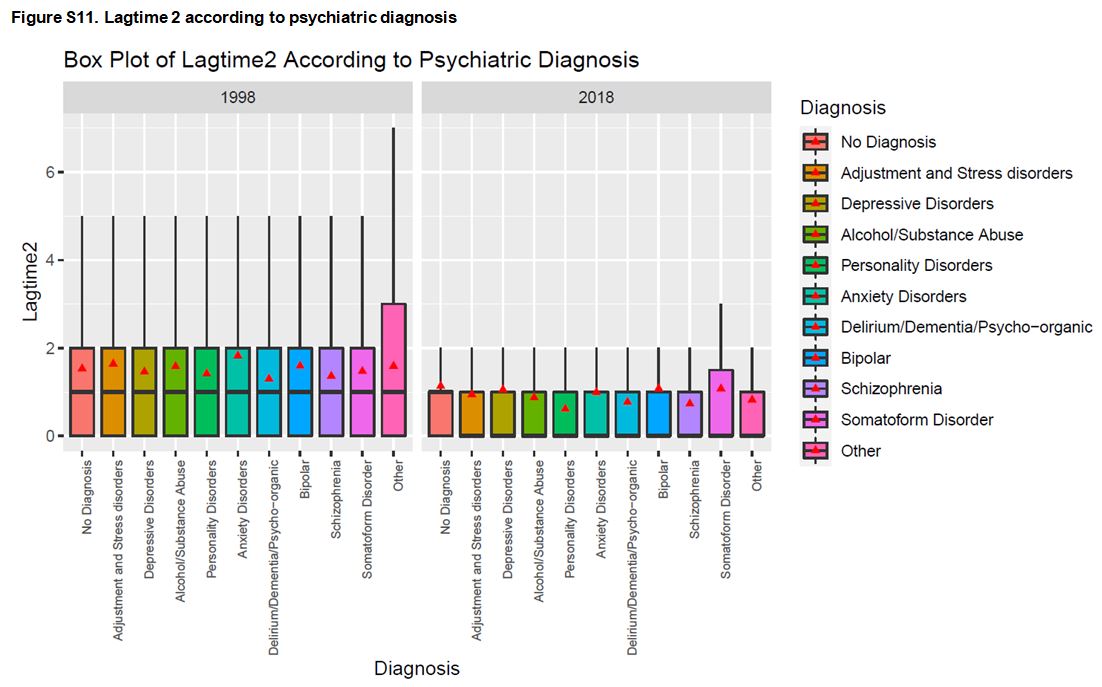

Supplement: Supplementary file 1 [file Data_Sheet_1.ZIP › Figure S11.JPG]

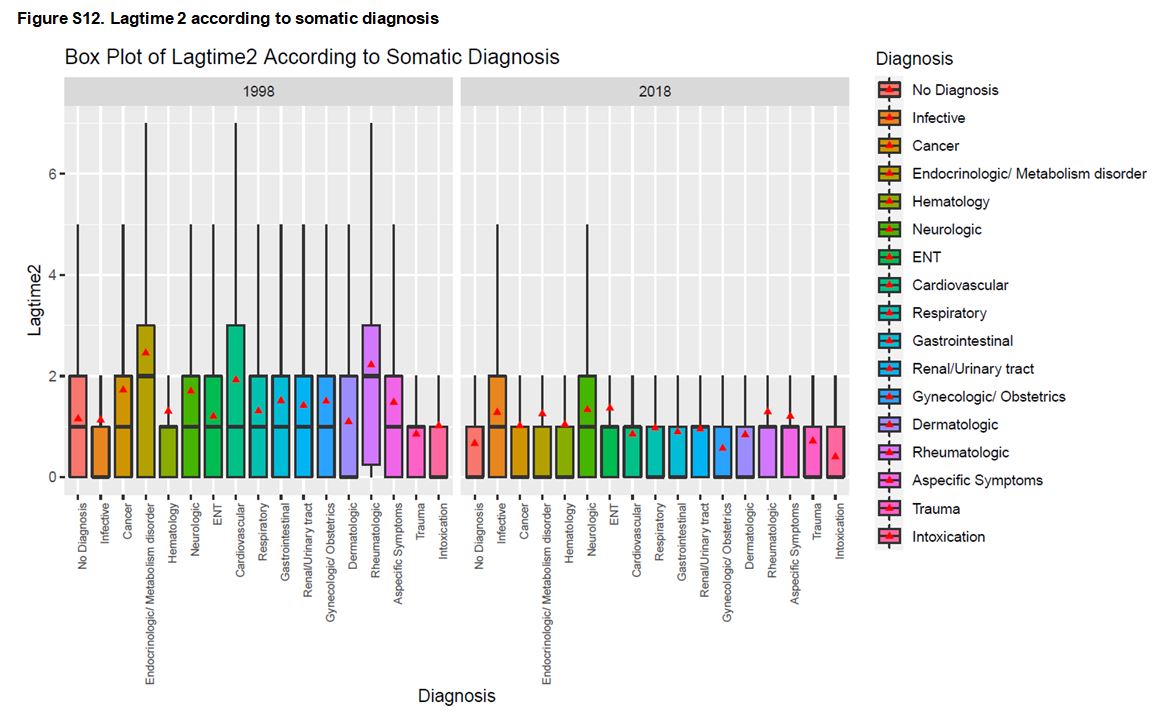

Supplement: Supplementary file 1 [file Data_Sheet_1.ZIP › FIgure S12.JPG]

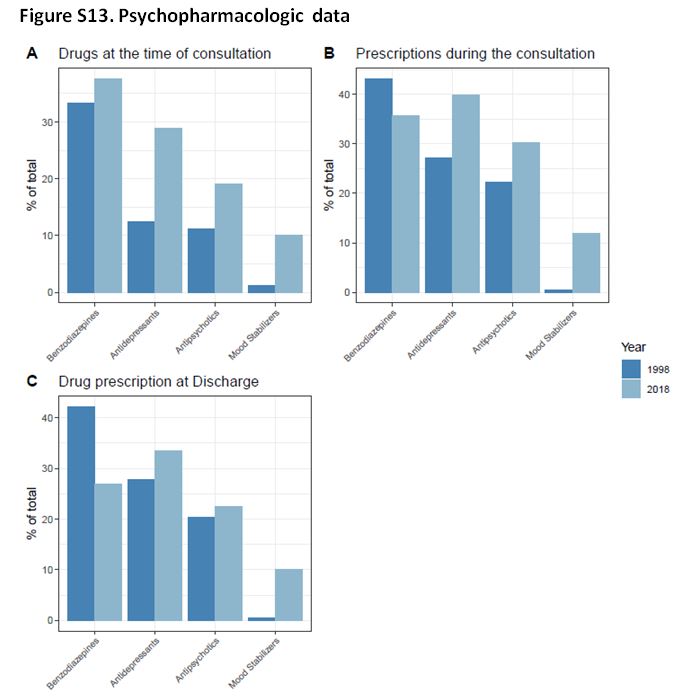

Supplement: Supplementary file 1 [file Data_Sheet_1.ZIP › figure S13 14.9.22.JPG]

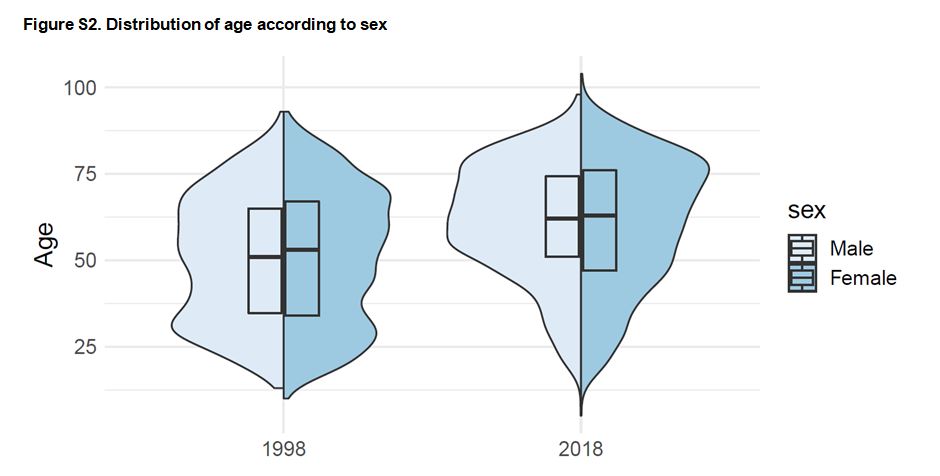

Supplement: Supplementary file 1 [file Data_Sheet_1.ZIP › Figure S2.JPG]

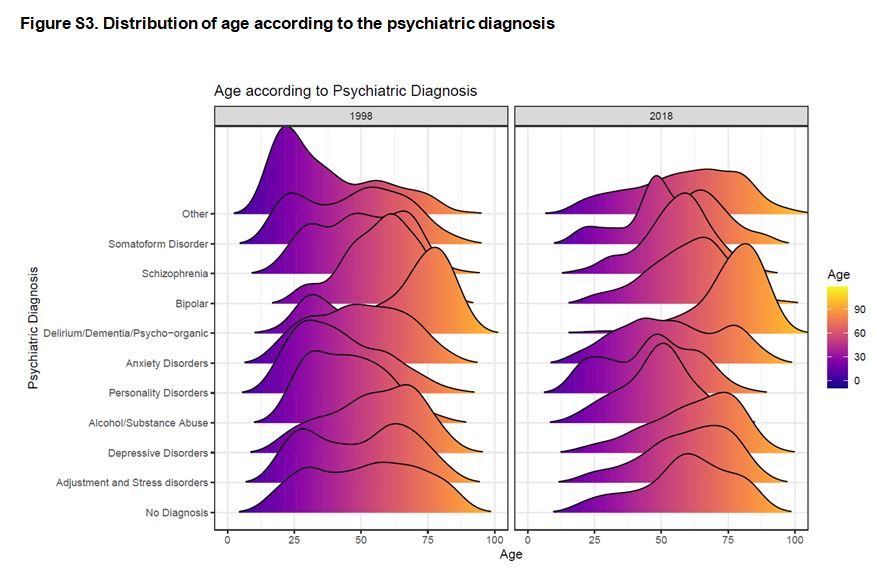

Supplement: Supplementary file 1 [file Data_Sheet_1.ZIP › Figure S3.JPG]

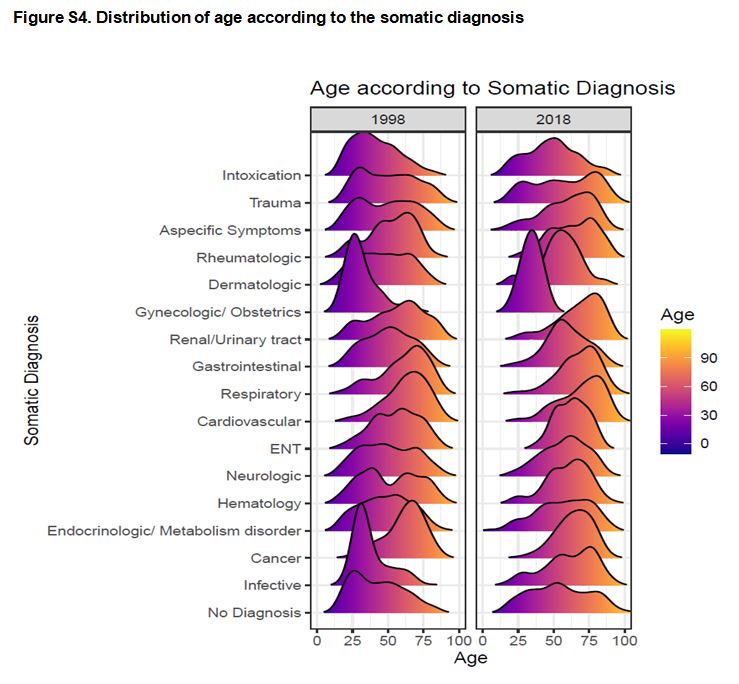

Supplement: Supplementary file 1 [file Data_Sheet_1.ZIP › Figure S4.JPG]

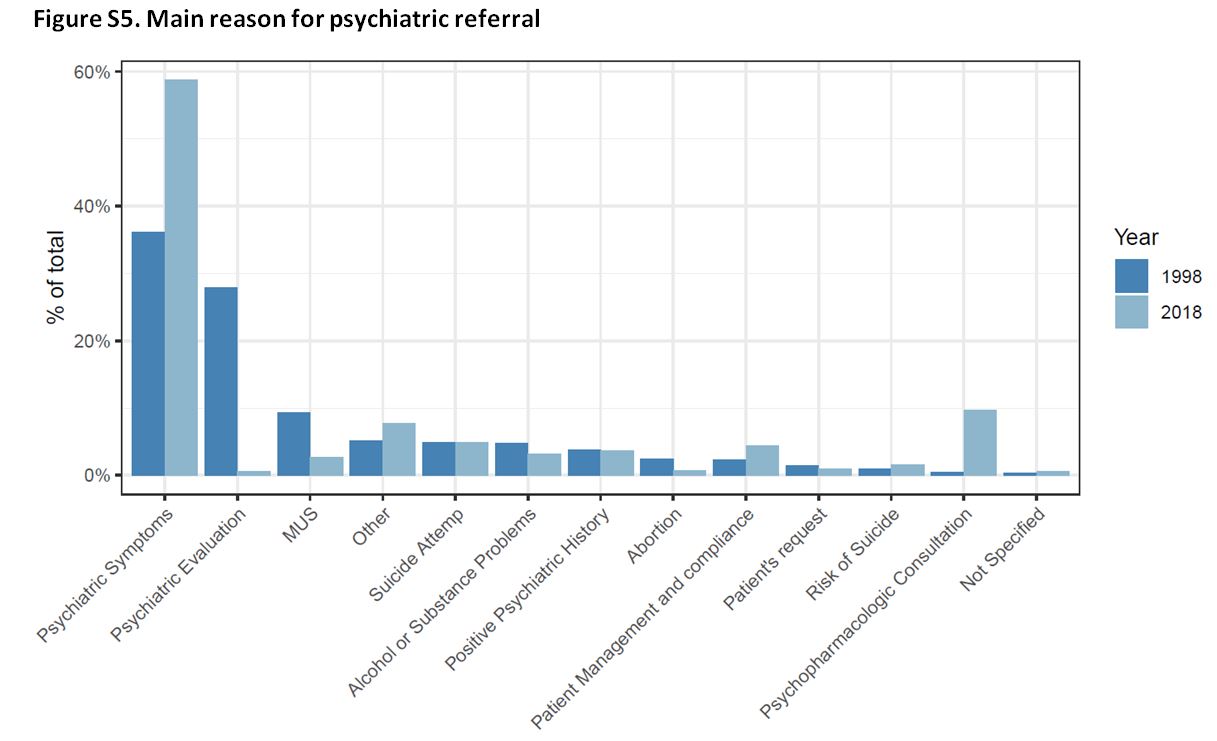

Supplement: Supplementary file 1 [file Data_Sheet_1.ZIP › figure s5 14.9.22.JPG]

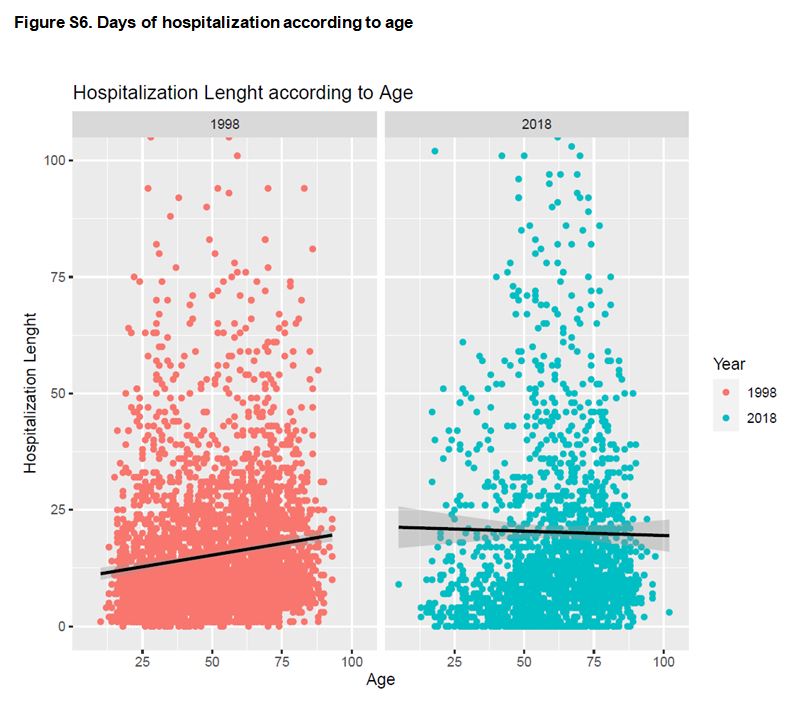

Supplement: Supplementary file 1 [file Data_Sheet_1.ZIP › FIgure S6.JPG]

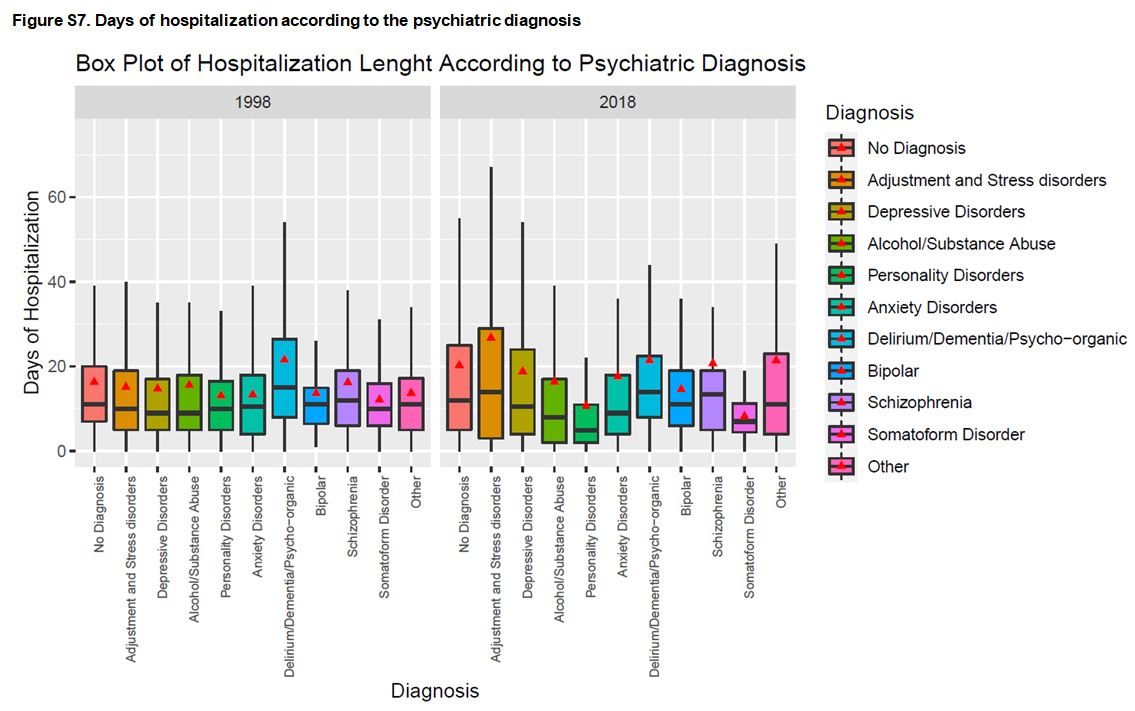

Supplement: Supplementary file 1 [file Data_Sheet_1.ZIP › Figure S7.JPG]

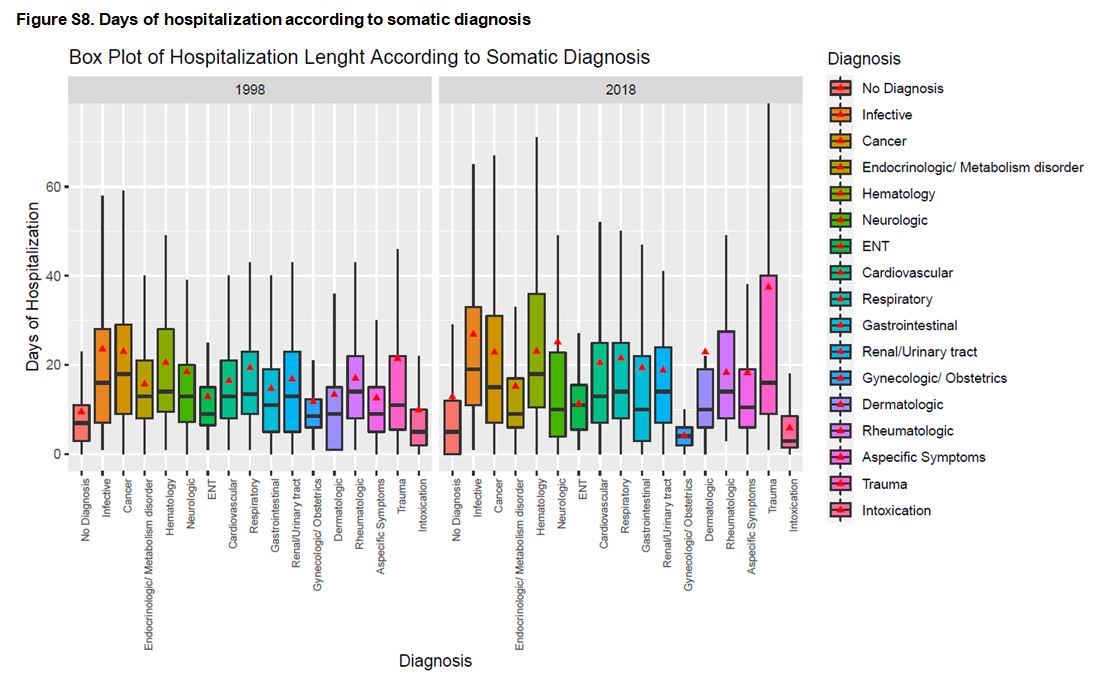

Supplement: Supplementary file 1 [file Data_Sheet_1.ZIP › FIgure S8.JPG]

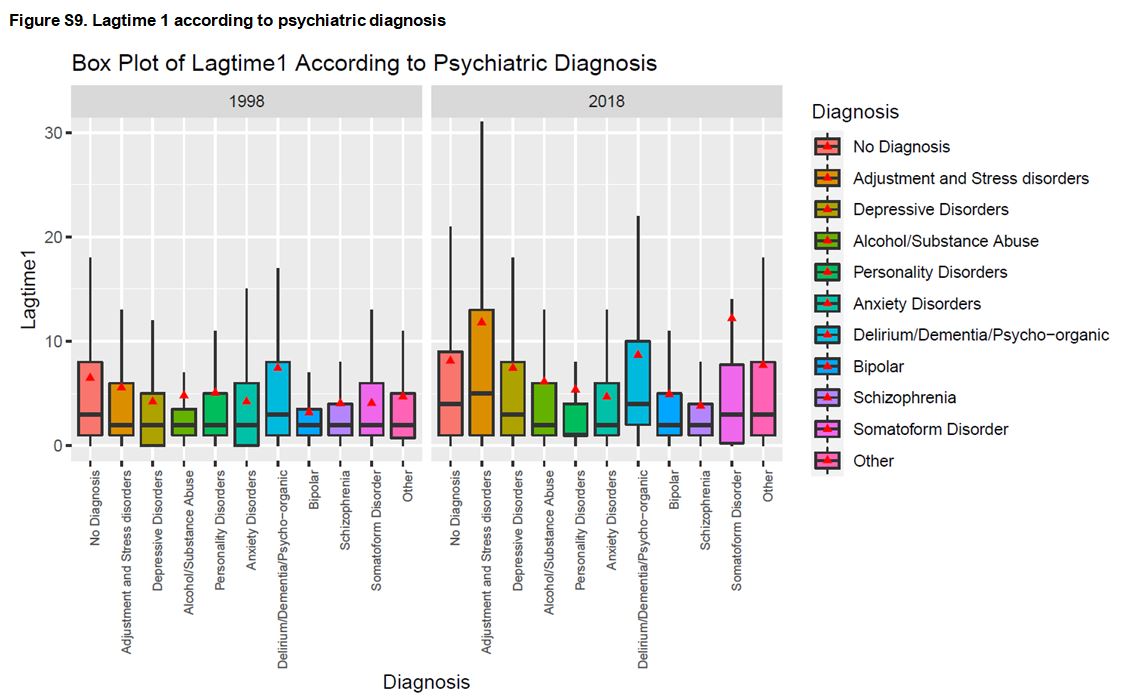

Supplement: Supplementary file 1 [file Data_Sheet_1.ZIP › Figure S9.JPG]
